# Neuronal Correlates of Individual Differences in the Big Five Personality Traits: Evidences from Cortical Morphology and Functional Homogeneity

**DOI:** 10.3389/fnins.2017.00414

**Published:** 2017-07-18

**Authors:** Ting Li, Xu Yan, Yuan Li, Junjie Wang, Qiang Li, Hong Li, Junfeng Li

**Affiliations:** ^1^Department of Radiology, Heping Hospital of Changzhi Medical College Shanxi, China; ^2^Department of Health Care, Changzhi Medical College Shanxi, China; ^3^Department of Psychiatry, First Hospital of Shanxi Medical University Shanxi, China

**Keywords:** personality traits, resting-state fMRI, cortical morphology, regional homogeneity, general linear model

## Abstract

There have been many neuroimaging studies of human personality traits, and it have already provided glimpse into the neurobiology of complex traits. And most of previous studies adopt voxel-based morphology (VBM) analysis to explore the brain-personality mechanism from two levels (vertex and regional based), the findings are mixed with great inconsistencies and the brain-personality relations are far from a full understanding. Here, we used a new method of surface-based morphology (SBM) analysis, which provides better alignment of cortical landmarks to generate about the associations between cortical morphology and the personality traits across 120 healthy individuals at both vertex and regional levels. While to further reveal local functional correlates of the morphology-personality relationships, we related surface-based functional homogeneity measures to the regions identified in the regional-based SBM correlation. Vertex-wise analysis revealed that people with high agreeableness exhibited larger areas in the left superior temporal gyrus. Based on regional parcellation we found that extroversion was negatively related with the volume of the left lateral occipito-temporal gyrus and agreeableness was negatively associated with the sulcus depth of the left superior parietal lobule. Moreover, increased regional homogeneity in the left lateral occipito-temporal gyrus is related to the scores of extroversion, and increased regional homogeneity in the left superior parietal lobule is related to the scores of agreeableness. These findings provide supporting evidence of a link between personality and brain structural mysteries with a method of SBM, and further suggest that local functional homogeneity of personality traits has neurobiological relevance that is likely based on anatomical substrates.

## Introduction

Personality is what makes every human unique, as it donates individual differences in behaviors, cognition, and emotion, which is stable over time and across situation. It has been assumed to have a neurobiological basis (Nostro et al., 2017), and the search for the neuronal foundation of human personality has guided research for decades.

The “five-factor model” or the “big five” is the most widely accepted taxonomies of personality (Costa and McCrae, 1992; Funder, 2001; Hu et al., 2011), representing tendencies to various aspects of social behavior and emotional stimulus: neuroticism, extroversion, openness, agreeableness, and conscientiousness. Neuroticism reflects dimensions of negative emotions, includes traits, such as anxiety, vulnerability, and irritability (Costa and McCrae, 1992; Clark and Watson, 2008). Extroversion is linked to the tendency to experience positive emotions, encompasses traits, such as assertiveness, sociability, and talkativeness (Costa and McCrae, 1992; Clark and Watson, 2008). Openness appears to reflect a preference for novelty and flexibility, which may be captured as intellectual curiosity and interests. Agreeableness tends to indicate the collection of traits related to altruism, people high on which tend to be helpful, sympathetic, and cooperative. Lastly, conscientiousness is manifested as impulsivity, orderliness, and self-discipline (De Young et al., 2010). The Neuroticism Extroversion Openness Five-Factor Inventory (NEO-FFI) designed by Costa and McCrae is widely used to explore the big five personality traits (Costa and McCrae, 1992).

Neuroimaging studies provided supporting evidences by demonstrating that inter-individual in personality traits are related with brain structures. For example, one study has shown that extroversion is associated with the medial orbitofrontal cortex, which is related to processing reward information, and that agreeableness is associated with several gray matter regions, such as the superior temporal sulcus and posterior cingulate cortex, which are related to social information processing (De Young et al., 2010). Another study has shown that extroversion is associated with the inferior frontal gyrus (Bjørnebekk et al., 2013). These studies have shed light on the correlation between personality traits and brain structures, however, most of these studies adopt a method of voxel-based morphology (VBM) analysis, which has been considered neglected the intrinsic geometry of the highly folded human cortex. Therefore, in the present research we conducted a surface-based analysis (SBM), which provides better alignment of cortical landmarks to explore whether structural variability on the cortical surface is related to the specific personality traits (Ghosh et al., 2010; Winkler et al., 2010; Mills and Tamnes, 2014). Moreover, recent work has successfully related two different SBM analyses: vertex-wise and regional parcellation–based. Vertex-wise analysis has been used to demonstrate the relationship between personality and the brain's structural and functional mechanisms (De et al., 2013; Chen et al., 2015); while using a regional parcellation approach, FreeSurfer (Destrieux atlas) can automatically segment the brain into different cortical regions of interest, and calculate average thickness—along with other closely related measures, such as surface area and volume—in the defined regions (Destrieux et al., 2010), and this automated method also has been used to some personality studies (Guadalupe et al., 2014). But combining vertex-wise and regional parcellation investigations with respect to morphology-personality relationships are still lacking. Here, we analyzed correlates of the morphology-personality relationships from two different levels in 120 healthy subjects within a large age range (18–60 years).

Regarding correlations between personality traits and brain structure, recent neuroimaging studies have reported that individual differences in vertex-wise regional homogeneity (2dReHo) are associated with those of cortical morphology (Jiang et al., 2015). It is known that regional homogeneity (ReHo) depended on the structural definition of nearest neighbors across the cortical mantle is believed to reflect synchrony of time series of neighboring voxels (Zuo et al., 2013) and such neighboring node information usually reflects anatomical, morphological, and intrinsically geometric features in local brain structure (Jiang and Zuo, 2016). Therefore, we examined 2dReHo analysis within regions identified in the regional SBM correlation, to further illustrate the functional homogeneity associated with neuroanatomical variations. Considering 2dReHo's high test–retest reliability and biological meaning (Zuo et al., 2013; Jiang et al., 2015), this approach can shed further light on whether 2dReHo relates to personality traits has neurobiological relevance that is based on anatomical substrates.

Thus the purpose of this study was to investigate whether significant correlations between personality traits and cortical morphology in healthy adults exist and also to investigate whether the regions identified in the regional SBM analysis show significant 2dReHo differences. Parameters of investigation include measures of total and regional brain volumes, cortical thickness, arealization, sulcus depth and mean curvature. Based on previous studies, we in general hypothesized negative correlations between brain structure and neuroticism, including decreasing thickness, surface arealization, brain volume, sulcus depth and mean curvature. Furthermore, there is some support linking extraversion to the volume of temporal cortex (Canli et al., 2001; Kapogiannis et al., 2013). Thus, for extraversion strongest relationships were expected in temporal regions. In conclusive findings related to the remaining three traits suggest that strong hypothesis regarding brain correlates of agreeableness, openness and conscientiousness would be premature and speculative. Additionally, we examined whether personality traits are related to 2dReHo within regions identified in the regional SBM analysis, to highlight the potential relations between regional structural variability and spatial local homogeneity.

## Experimental procedure

### Participants

MRI scans were acquired for 120 right-handed healthy adults (50 males, range 19–60). Most participants were recruited from Shanxi medical university or advertisements on bulletin boards in the community. Twelve participants were excluded because of the incomplete MRI or questionnaire data. Therefore, the final sample consisted of 108 participants (age range: 19–60 years; 44 males). All participants had no history of psychiatric or neurological illness, cognitive disability, substance abuse (including illicit drugs and alcohol). After providing written informed consent and MRI scans, participants were required to undertake a series of psychological tests, and they subsequently received payment for their time. Subjects signed the informed consent prior to the experiment on the premise of fully understanding the content of the experiment. The Ethics Committee of the Shanxi Medical University approved this study.

### Assessment of personality

The Neuroticism Extroversion Openness Five-Factor Inventory (NEO-FFI) was designed by Costa and McCrae (1992), consists of 60 questions on a 5-point scale. These questions measure personality across five domains: Neuroticism (N), Extroversion (E), Openness (O), Agreeableness (A), and Conscientiousness (C). This simplified version is proven to be consistent with the full version, and has good convergent validity with other personality scales (Costa and McCrae, 1989; Parker and Stumpf, 1998; Kurtz and Sherker, 2003).

### Data acquisition

All MRI images were collected using a 3.0 T SIEMENS Trio scanner at Shanxi Provincial People's Hospital. Structural MRI images were acquired using a 3D magnetization-prepared rapid gradient-echo (MPRAGE) T1-weighted sequence (*TR* = 2,300 ms; *TE* = 2.95 ms; *TI* = 900 ms; flip angle = 9°, FOV = 225 × 240 mm, 160 slices, thickness = 1.2 mm). And the resting-state fMRI image: echo planar imaging (EPI) pulse sequence (32 slices, *TR* = 2,500 ms; *TE* = 30 ms; *FA* = 90°, matrix = 64 × 64, FOV = 240 × 240 mm, 160 slices, thickness = 4 mm, and 212 volumes).

### Image preprocessing

Image preprocessing was carried out using the Connectome Computation System (CCS: http://lfcd.psych.ac.cn/ccs.html) (Zuo et al., 2013); —an integration system that involves AFNI, FSL, Freesurfer (Cox, 2012; Fischl, 2012; Jenkinson et al., 2012), and in MATLAB scripts for multimodal image analysis for discovery brain sciences (Sporns, 2015). The structural image preprocessing primarily included (1) denoising the structural image by means of a spatially adaptive non-local means filter (Zuo and Xing, 2011), (2) reconstruction of cortical surface, (3) segmentation of the cerebrospinal fluid (CSF), whitematter (WM), and graymatter (GM) volumetric structures, (4) estimation of a triangular mesh tessellation over the GM-WM boundary and the mesh deformation to produce a smooth representation of the GM-WM interface (white surface) and the GM-CSF interface (pial surface) spatial normalization from individual native space to fsaverage stereotaxicspace, (5) correction of topological defect on the surface, (6) inflation of individual surface mesh into a sphere, and (7) estimation of the deformation between the resulting spherical mesh and a common spherical coordinate system (Li et al., 2014). The functional image preprocessing included (1) drop of the first five volumes, (2) slice timing correction, (3) 3D motion correction, (4) 4D global mean-based intensity normalization, (5) nuisance regression (the WM and CSF mean time series and the Friston-24 motiontime series) (Yan et al., 2013), (6) band-pass filtering (0.01–0.1 Hz), (7) removal of linear and quadratic trends, (8) coregistration between individual structural and functional images by the GM-WM boundary-based registration (BBR) algorithm (Greve and Fischl, 2009), and (9) projection of the individual preprocessed 4D RFMRI time series onto a standard cortical surface fsaverage5.

Various figures and indices were produced to ensure the quality of processed images. The structural data quality control procedure (QCP) was performed by two researchers, included visual head motion inspection, tissue segmentation, and brain surface reconstruction. It was especially important to visually assess the quality of the brain extraction and to correct intensity bias to select the best images from the three alternative maps. The QCP of functional images included the warp distortion amount for BBR-based function-to-structure realignment as measured by the minimal cost of the head motion as measured by the root mean square of frame-wise displacement.

### Surface-based morphology analysis

Surface-based morphology analyses (SBM) of cortical indicators (thickness, surface area, volume, mean curvature, and sulcus depth) were performed using the CCS. These separate indices were estimated to measure different properties of brain cortical surface morphology. Cortical thickness is the averaged linking distance between the pial and white surfaces along normal vector (Fischl and Dale, 2000). Surface area is the total area of the triangles that were connected to a vertex (Fischl and Dale, 2000). Volume is quantified by cortical thickness and surface area. Given a vertex on the cortical surface, its mean curvature is the mean of the two principal curvatures, which measure the maximum and minimum bending of the cortical surface at that vertex (Pienaar et al., 2008). Sulcus depth is defined as the product of the displacement and regular unit vector in the process of the expansion of the cortex, used for quantifying the large scale geometric information of cortex (Jiang et al., 2015).

### Surface-based ReHo analysis

Kendall's coefficient of concordance (KCC) was used to measure ReHo of the time series of a given voxel with its nearest neighbors in a voxel-wise fashion (Zang et al., 2004). Vertex-wise functional homogeneity analysis was performed with CCS on the cortical surface by adopting the classic ReHo method to its 2-dimensional variant (Zuo et al., 2013). The individual preprocessed 4D RFMRI time series were initially projected onto the fsaverage5 standard cortical surface for determining the vertex of the surface space. To calculate 2dReHo, for a given vertex, lengthone has six neighbor vertices in the surface space, and the KCC of rsfMRI from seven vertexes (including the given vertex) was computed (Zuo et al., 2013). This computational procedure was repeated for all vertices in surfaces of both hemispheres to produce vertexwise KCC-ReHo surface maps.

### Statistical analysis

A general linear model (GLM) was used to examine the relationship between morphometric dimensions on the brain surface and personality traits. The effect of gender, age, and the intracranial volume (ICV) as covariates were regressed out. The significance level was set at 5% for each of the independent regressions, corrected for multiple comparisons using false discovery rate (FDR).

To further illustrate local features of the regional parcellation, linear regressions were calculated with Matlab-based functions to examine the relationships between regional homogeneity and personality trait scores. We first selected a region on the basis of the regional-based morphology analysis, after that we extracted the region's average functional indicators (2dReHo) for each subject. 2dReHo was entered into regressions to predict personality scores after controlling for sex, age and ICV, corrected for multiple comparisons using FDR.

## Results

### Behavioral results

**Figure 1:**
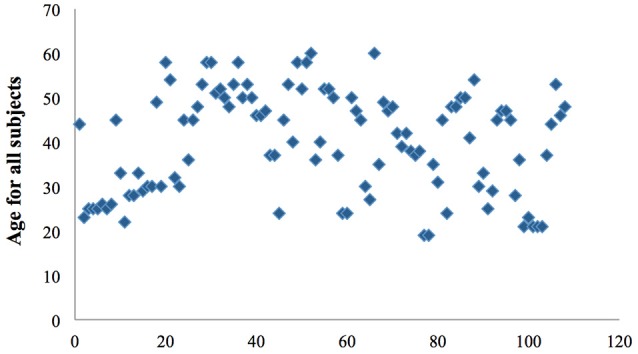
Scatter diagram shows the age distribution of all the final subjects.

Table 1 shows the descriptive statistics for the demographic and psychological characteristics of all participants (*N* = 108; males = 44, females = 64). Figure 1 shows the age distribution of the subjects.

**Table 1:** Descriptive statistics for the demographic and psychological characteristics.

**Measure**	**Mean**	**SD**	**Range**
Age	40.28	11.43	19–60
Years of education	14.49	2.80	9–20
Neuroticism(N)	31.78	6.30	16–49
Extraversion(E)	39.40	6.78	20–56
Openness(O)	32.67	4.26	28–50
Agreeableness(A)	42.07	3.80	31–51
Conscientiousness(O)	42.30	6.11	24–58

### Vertex-wise morphology analysis.

**Figure 2:**
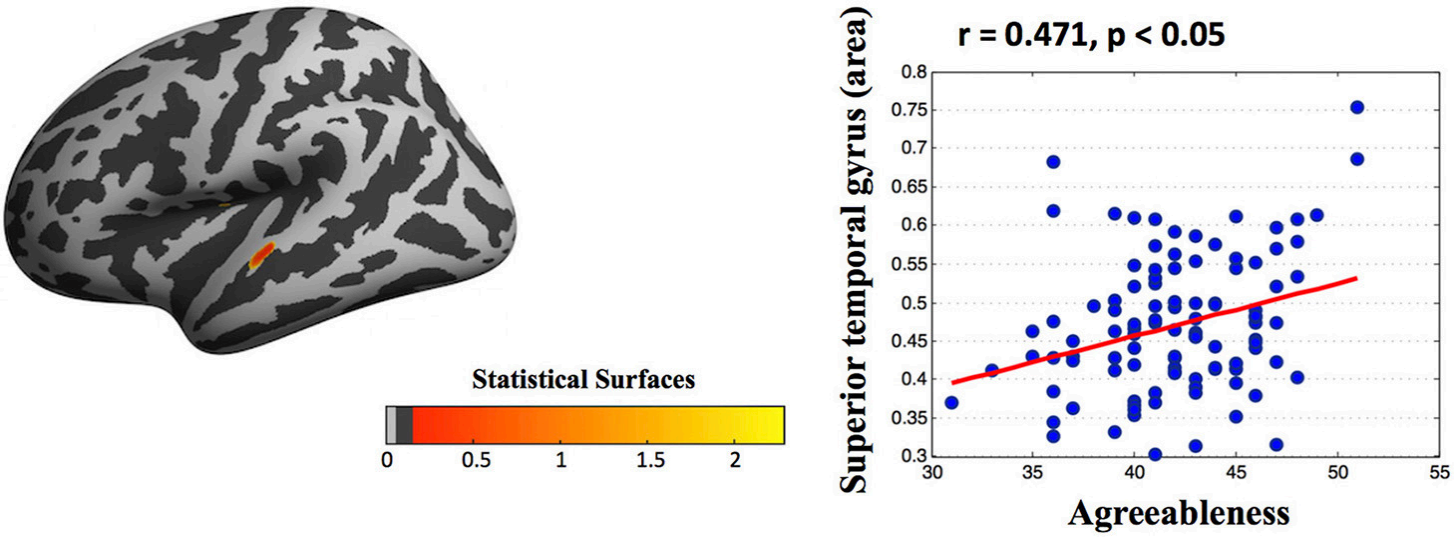
Statistical maps of cortical surfaces showing a significant correlation (FDR corrected) between agreeableness and area measured by surface-morphology analysis based on vertex-wise in fsaverage standard space. After controlling for age, sex, and ICV, a multiple regression analysis found that high agreeableness scores were associated with large areas in the superior temporal gyrus. Note that this scatter plot is presented only for the purpose of visualization.

Vertex-wise morphology analysis showed an association between the scores of personality and cortical indexes. After controlling for age, sex, and ICV, a multiple regression analysis revealed that higher agreeableness was associated with larger areas in the superior temporal gyrus (peak: x, y, z = −61.51, −15.92, −1.14; Figure 2).

### Regional-based morphology analysis

**Figure 3:**
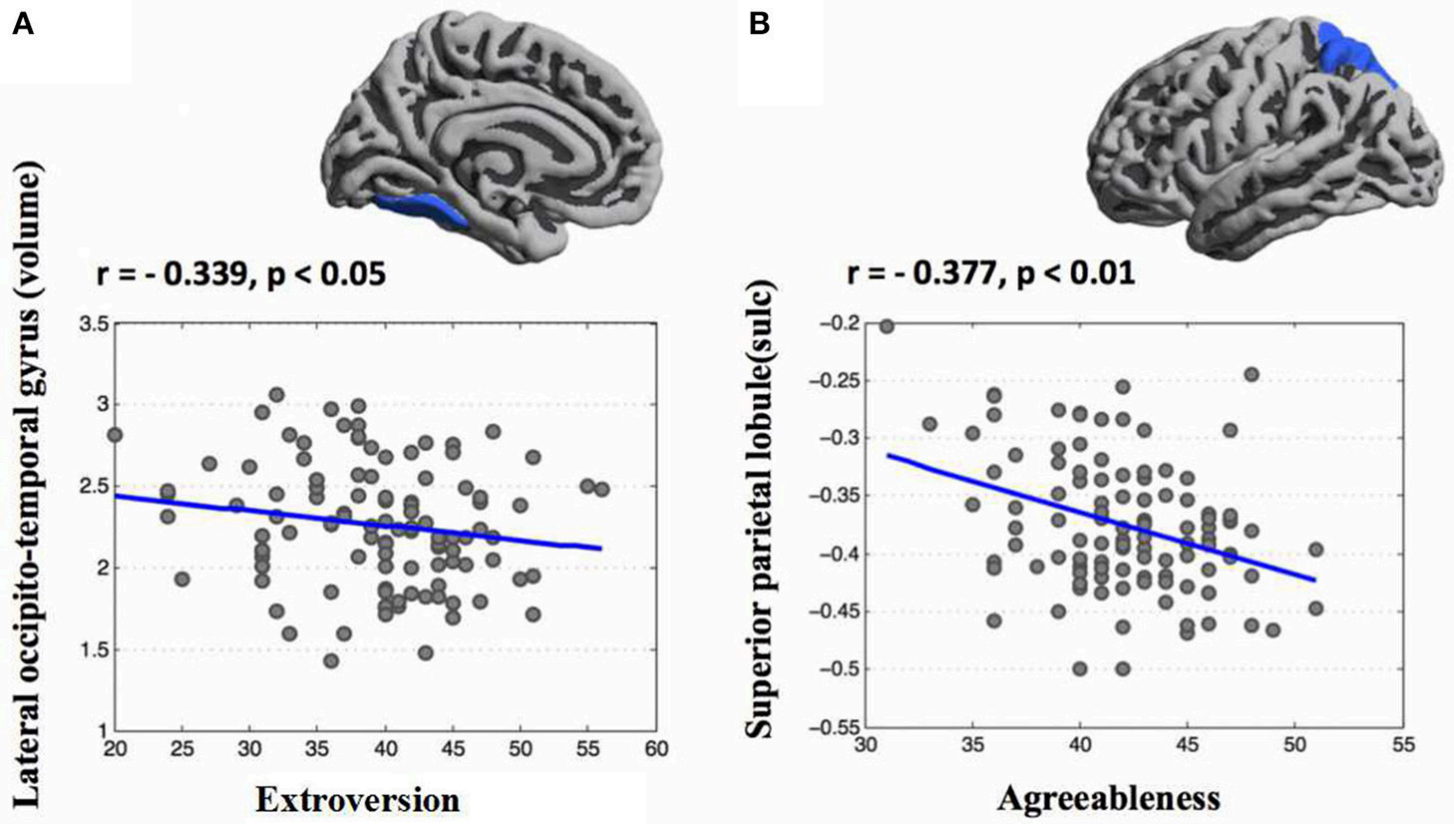
Correlation maps of regional parcellation between personality traits and morphometric features (FDR corrected). After controlling for age, sex, and ICV, a multiple regression analysis found that extroversion was negatively related with the volume of the left lateral occipito-temporal gyrus **(A)** and agreeableness was negatively associated with the sulcus depth of the left superior parietal lobule **(B)**.

Based regional parcellation we examined the association between personality scores and cortical indicators. After controlling for age, sex, and ICV, a multiple regression analysis revealed extroversion was negatively related with the volume of the left lateral occipito-temporal gyrus and agreeableness was negatively associated with the sulcus depth of the left superior parietal lobule (Figure 3). Additional statistical information concerning brain regions is provided in Table 2.

**Table 2:** Brain areas that are significantly correlated with personality traits and cortical indicators.

**Personality trait**	**Morphometric parameters**	**Brain region**	**Cortical hemisphere**	***t***
Extraversion	Volume	Lateral occipito-temporal gyrus	Left	−3.860^*^
Agreeableness	Depth of sulcs	Superior parietal lobule	Left	−4.167^**^

* *Indicates p < 0.05*.

** *Indicates p < 0.01(corrected)*.

### Regional-based functional homogeneity analysis.

We also performed regional functional homogeneity analysis by converting fsaverage to fsaverage5 standard space, extracting each subject's average 2dReHo in the significant regions, which was identified in regional-based morphology measures, and then tested the linear relationship between 2dReHo and corresponding personality traits, controlling for sex, age, and ICV. Subjects who had high scores for extroversion had higher regional homogeneity in the left lateral occipito-temporal gyrus (*r* = 0.299, *p* < 0.01), subjects who had high scores for agreeableness had higher regional homogeneity in the left superior parietal lobule (*r* = 0.181, *p* < 0.05; see Table 3).

**Table 3:** Correlations of regional parcellation between personality traits and functional homogeneity.

**Brain region**	**Personality trait**	**Cortical hemisphere**	***t***
Lateral occipito-temporal gyrus(L)	Extraversion(NEO)	Left	3.091^**^
Superior parietal lobule(L)	Agreeableness(NEO)	Left	−2.083^*^

* *Indicates p < 0.05*.

** *Indicates p < 0.01(corrected)*.

## Discussion

This study revealing the differences in the relationship between brain morphology and personality traits on two different levels: vertex-wise and regional parcellation, and two methods obtained results are not overlay. Part of the variations may be due to the use of different methodologies. Whole-brain analysis applies a method for calculation of the correlation between each vertex and personality traits, while using regions defined by the Destrieux atlas conducted a method for calculation of the mean value of the regional vertexes. Consequently, findings using such different approaches establish their robustness. On the other hand, if one of the techniques is more sensitive than the other, subtle cortical changes could simply be missed by one of the methods. Relatively speaking vertex-wise analysis may give a more accurate representation of structural differences because it takes account the change of each vertex. While it has been pointed out that a relationship between reliability and region size exists (Tustison et al., 2014), and using region-based analysis may have higher test-retest reliability. This may explain some of the discrepancies between the results of our two studies.

Based on vertex-wise analysis, we showed a significant positive correlation between the surface area of the superior temporal gyrus (STG) and agreeableness. The STG is involved in the interpretation of other individuals' actions and intentions on the basis of biological motion (Pelphrey and Morris, 2006), a process that may be more efficient in individuals who score higher in agreeableness. Further, involvement of the STG in emotional processing and effective responses to social cues, such as facial expressions and eye direction (Singer, 2006; Pelphrey and Carter, 2008), is well established. A previous structural study reported bigger STG volume with higher agreeableness scores (Kapogiannis et al., 2013), and change in cortical volume in the human brain has been attributed mainly to change in surface area rather than change in thickness (Pakkenberg and Gundersen, 1997; Im et al., 2008). Moreover, a functional neuroimaging study had shown that agreeableness was primarily a dimension of interpersonal tendencies (Moll et al., 2005; Behrens et al., 2009), it would predict connectivity with regions subserving altruism and social information processing, including the occipital cortex and temporal cortex (Kober et al., 2008), adding to the hypothesis that structural and functional properties of STG reflect individual differences in agreeableness.

Based on regional parcellation, a negative relationship was found between extroversion and the volume of lateral occipito-temporal gyrus (OTG). Extroversion, a trait reflecting proneness to experience positive emotions and engage in social interactions (Canli et al., 2001; Lucas and Diener, 2001), was associated with larger cortical volume within dorsolateral PFC and temporal regions. Volume of temporal and occipital cortex associated with extroversion had been found in the previous study (Kapogiannis et al., 2013), and our study provides further evidence for this association. Meanwhile, we also found that decreased sulcus of superior parietal lobule (SPL) was associated with agreeableness. Similarly, there were reports that structural variation in superior parietal is considered to be associated with higher agreeableness scores (Kapogiannis et al., 2013). Previous structural neuroimaging studies in psychopathology had revealed that individual with cluster B personality disorders had been observed structural alteration of superior parietal cortices, suggesting a possible role of parietal cortex in the integration of many emotional and cognitive functions, such as sensory information and processing (part of the features of agreeableness), this implied the possibility that agreeableness may be, at least, partly related with SPL (Irle et al., 2005, 2007).

Specifically, to further interpret the regional findings we tested the linear relationship between 2dReHo in the significant regions, which was identified in regional-based morphology analysis and the corresponding personality traits. A positive relationship was found between extroversion and 2dReHo of the left lateral OTG, agreeableness and 2dReHo of the left SPL. We indeed found that joint variations in anatomical features and synchronized spontaneous fluctuations on the brain surface strongly predicted individual personality. In a similar vein, Chen et al. (2015) reported that people with high verbal creative ability exhibited lower regional functional homogeneity in the right precuneus, and both cortical volume and thickness of the right precuneus were positively associated with individual verbal creativity (Chen et al., 2015). These findings provide evidence for a relationship between structural plasticity and intrinsic architecture of the brain. Additionally, study findings support the assertion that ReHo not only holds its own unique functional variability but also shares the individual variability with a wide range of cortical morphologies (Jiang et al., 2015). In sum, our findings may provide important insights into how brain affects personality traits, although the nature of this structure-function relationship is complex and should be further investigated.

This study has some limitations. The first, this study found only with extroversion and agreeableness related brain structure mechanism, and did not find the neurobiological mechanism of other three personality traits. The reason may be due to the characteristics of the subjects and the future research needs to use a larger sample size to explore the brain structure of personality traits. Secondly, this is a cross-sectional study, in order to further understand the brain structural changes associated with the personality, longitudinal studies are still needed to examine. Lastly, although local functional homogeneity of personality traits might have an anatomical basis, the mechanism of the relationship or the existence of interaction effects between structure and function remains unknown. Future research should explore the joint contribution of structural and functional brain networks in personality traits using diffusion weighted imaging and resting-state fMRI data.

In summary, the findings of present study demonstrate that individuals with high agreeableness scores had larger areas in the superior temporal gyrus. Based on regional parcellation, we found that extroversion was negatively related with the volume of the left lateral OTG and agreeableness was negatively associated with the sulcus depth of the left SPT. Additionally, increased regional homogeneity in the left lateral OTG is related to the scores of extroversion and the increased regional homogeneity in left SPT is related to the scores of agreeableness. These observations suggest that individual differences in functional heterogeneity affected by brain structure might facilitate individual personality traits. The results provide additional evidence of the associations between brain structure and variations in personality by using surface-based analysis, and support that regional heterogeneity markers reflect the cortical morphology organization of the brain and neurodevelopmental factors.

## Ethics statement

This study was carried out in accordance with the recommendations of the principle of voluntariness, the Shanxi Medical University with written informed consent from all subjects. All subjects gave written informed consent in accordance with the Declaration of Helsinki. The protocol was approved by the Ethics Committee of the Shanxi Medical University.

## Author contributions

HL and JL designed and supervised the study. TL, XY, and YL drafted the manuscript. JW carried out the experimental procedures. JW and QL participated in data processing.

### Conflict of interest statement

The authors declare that the research was conducted in the absence of any commercial or financial relationships that could be construed as a potential conflict of interest.
